# Multiple Stability Mechanisms Act Independently or in Concert to Maintain the Temporal Stability of Natural Communities

**DOI:** 10.3390/plants15081143

**Published:** 2026-04-08

**Authors:** Zhenyuan Duan, Zhihong Zhu

**Affiliations:** College of Life Sciences, Shaanxi Normal University, No. 620, West Chang’an Avenue, Chang’an District, Xi’an 710119, China; duanzhenyuan0704@snnu.edu.cn

**Keywords:** clipping, fertilization, stability mechanisms, temporal stability

## Abstract

The maintenance mechanisms underlying community temporal stability represent a pivotal concern in ecology. However, empirical evidence on how multiple mechanisms independently or synergistically stabilize natural communities, and how their importance responds to external factors and evolves over time, remains limited. Leveraging a 12-year (2007–2018) manipulative experiment involving clipping and fertilization in an alpine meadow, we assessed the relative contributions of four mechanisms, namely, species asynchrony (compensatory dynamics among species), the portfolio effect (statistical averaging of species’ fluctuations), the selection effect (dominance of stable species), and interspecific interactions, across treatments and temporal scales. Stability was quantified as the reciprocal of the coefficient of variation in community coverage. Asynchrony was a ubiquitous foundation of stability across all treatments and time periods. The portfolio effect was a critical positive driver in the initial phase but was suppressed by fertilization over time. In contrast, interspecific interactions and the selection effect emerged as central determinants of long-term stability in later stages. Fertilization amplified the portfolio effect and fostered weak interactions while reducing the fluctuation disparity between dominant and non-dominant species. Clipping enhanced stability mechanisms by preserving species richness and asynchrony. Structural equation modelling revealed that treatments indirectly influenced stability by “reprogramming” the causal pathways among these mechanisms. Our study demonstrates that community stability is upheld by multiple coordinated mechanisms, whose relative importance is contingent on treatment and time scale. Grassland management should therefore move beyond a singular focus on species richness and adopt strategies that promote the synergistic functioning of multiple stability mechanisms.

## 1. Introduction

The mechanisms underlying community temporal stability represent a fundamental topic in ecology [1,2], with critical implications for understanding community stability and the maintenance of ecosystem functioning [3]. Temporal stability is defined as the ability of an ecosystem to maintain its functional integrity while resisting environmental disturbance [4], a property modulated by diverse biotic and abiotic factors [5]. Among the various metrics developed to assess ecosystem stability, particularly within biodiversity research, temporal stability is frequently quantified as the inverse of the coefficient of variation [6].

Both theoretical and empirical studies have substantiated the positive effects of biodiversity on ecosystem productivity and stability, namely, enhanced productivity and the capacity to resist disturbances and maintain functional integrity. At least six mechanistic hypotheses have been proposed to explain the maintenance of temporal stability in ecosystem functioning: (1) the asynchrony effect (asynchronous responses of species to environmental fluctuations) [2]; (2) the portfolio effect (statistical averaging of species’ variances) [7]; (3) the overyielding effect (increased total community biomass with greater diversity) [8]; (4) the selection effect (enhanced variability of total community biomass due to dominance by species with specific traits, which reduces ecosystem stability) [4]; (5) facilitation (direct positive interactions between species, often through the amelioration of environmental stress); (6) interspecific interactions (weak interactions, increased top–down consumer pressure transforming strong trophic competition among lower-level species into weak interactions, thereby reducing mutual interference and enhancing the temporal stability of populations and communities) [7,8,9,10,11,12,13,14,15,16,17,18,19,20,21].

Classical theory posits that diversity and complexity may destabilize communities. The relationship between biodiversity and ecosystem stability has long been a central and contested topic in ecology. Early theoretical work, most notably that of May [13], demonstrated that in model systems characterized by random interaction structures, increasing complexity, through a greater number of species and interspecific connections, can mathematically destabilize community dynamics. In such systems, complexity reduces the likelihood of returning to equilibrium following small perturbations. Conversely, a growing body of both theoretical and empirical research has revealed that biodiversity can enhance the temporal stability of ecosystem functions, such as aggregate biomass or productivity. This stabilizing effect is primarily achieved through mechanisms that reduce temporal variability, including species asynchrony and statistical averaging. However, such stability at the ecosystem level may come at the cost of increased variability in the abundances of individual component species [8,16]. Collectively, these contrasting perspectives suggest that whether diversity stabilizes or destabilizes a system depends critically on the type of stability under consideration and the specific ecological context in which it is assessed. In contrast, recent evidence suggests that biodiversity can enhance the overall stability of ecosystems or communities, albeit potentially at the cost of destabilizing the richness of individual species [8,16]. These mechanisms may operate interdependently (asynchrony effect, portfolio effect, overyielding, facilitation, and weak interactions) or independently of diversity, as is the case for the selection effect. Moreover, these processes may act in concert or in isolation. Despite this theoretical groundwork, the operation of these mechanisms in natural communities remains poorly resolved.

It is well established that the diversity and structural and functional complexity of natural ecosystems, as well as their ecological stability, are influenced by multiple factors such as resource availability and disturbance regimes. The ensuing shifts in interspecific interaction dynamics, in turn, determine which specific mechanism, or combination thereof, sustains ecosystem functional stability [20]. The asynchrony effect, for instance, arises from negative correlation in species’ responses to environmental fluctuations driven by interspecific competition and the complementarity effect, thereby conferring stability [14]. The portfolio effect posits that stability increases with species richness due to random fluctuations among species [18]. Weak interspecific interactions occur when enhanced top–down pressure alleviates interspecific interaction, transforming intense resource competition among lower trophic levels into attenuated trophic interactions; this shift damps population fluctuations and enhances community temporal stability. Collectively, these perspectives suggest that the dominant mode of interspecific interaction is a primary determinant of the principal stability mechanism operating within a community. Other mechanisms, while potentially present, may not contribute substantially to realized stability [18].

To date, the majority of empirical studies have relied on spatial regression models that implicitly assume that the observed patterns of temporal stability reflect variation in the equilibrium states of the ecological processes under investigation [2]. Such approaches inherently overlook the temporal dynamics of interspecific interaction regimes. Likewise, biodiversity manipulation experiments often presuppose static or invariant environmental conditions governing the focal ecological processes. However, if the nature of interspecific interactions shifts over time, both the level of stability and the mechanisms maintaining it are likely to change accordingly. Thus, the stability of natural ecosystem functions and the mechanisms that underpin it may be contingent upon resource availability, disturbance regime, and temporal context. For example, research conducted in Inner Mongolian grasslands has demonstrated that alterations in precipitation patterns and overgrazing intensity can profoundly modify ecosystem stability [19]. These findings underscore that, under the influence of interacting biotic and abiotic factors, the mechanisms sustaining functional stability in natural ecosystems are not static; rather, their temporal occurrence and relative importance vary dynamically.

The alpine meadow on the Tibetan Plateau is highly sensitive to climate change and human activities (such as fertilization and clipping), and exhibits high species turnover and interannual variability [5], providing an ideal natural platform for studying the dynamic changes in multiple stability mechanisms. Here, we report results from a 12-year (2007–2018) manipulative experiment investigating the effects of fertilization and clipping regimes on an alpine meadow ecosystem on the Tibetan Plateau. Leveraging long-term, spatial-scale empirical evidence on species richness, the dynamics of interspecific interactions, and temporal stability, we examined how the mechanisms maintaining temporal stability shift along temporal (within-community) and spatial (among-community) gradients, and evaluated how these shifts relate to varying clipping intensity and nitrogen enrichment levels. We first quantified the responses of relevant stability metrics to the experimental treatments over the full 12-year study period and assessed candidate stability mechanisms across the spatial gradient of communities. Subsequently, we partitioned the dataset into four sequential temporal subsets based on measured soil nitrogen content (Period I: 2007–2009; Period II: 2010–2013; Period III: 2014–2015; Period IV: 2016–2018), and estimated both the stability metrics and the underlying mechanisms operating during each interval. Finally, using structural equation modelling that integrates spatial and temporal dimensions, we identified the stability mechanisms operating within given communities across different periods, as well as those operating across different communities within the same period, and quantified their relative contributions to community stability. On the basis of the foregoing synthesis, we propose three key hypotheses: (1) the mechanisms maintaining functional stability in alpine meadow ecosystems are dependent on both resource availability and clipping intensity; (2) the expression of specific stability mechanisms is temporally dependent; and (3) the temporal stability of a given community is governed by the joint action of multiple mechanisms, yet the relative contributions of these mechanisms shift over time.

## 2. Results

### 2.1. Species Richness and Community Stability

**Figure 1:**
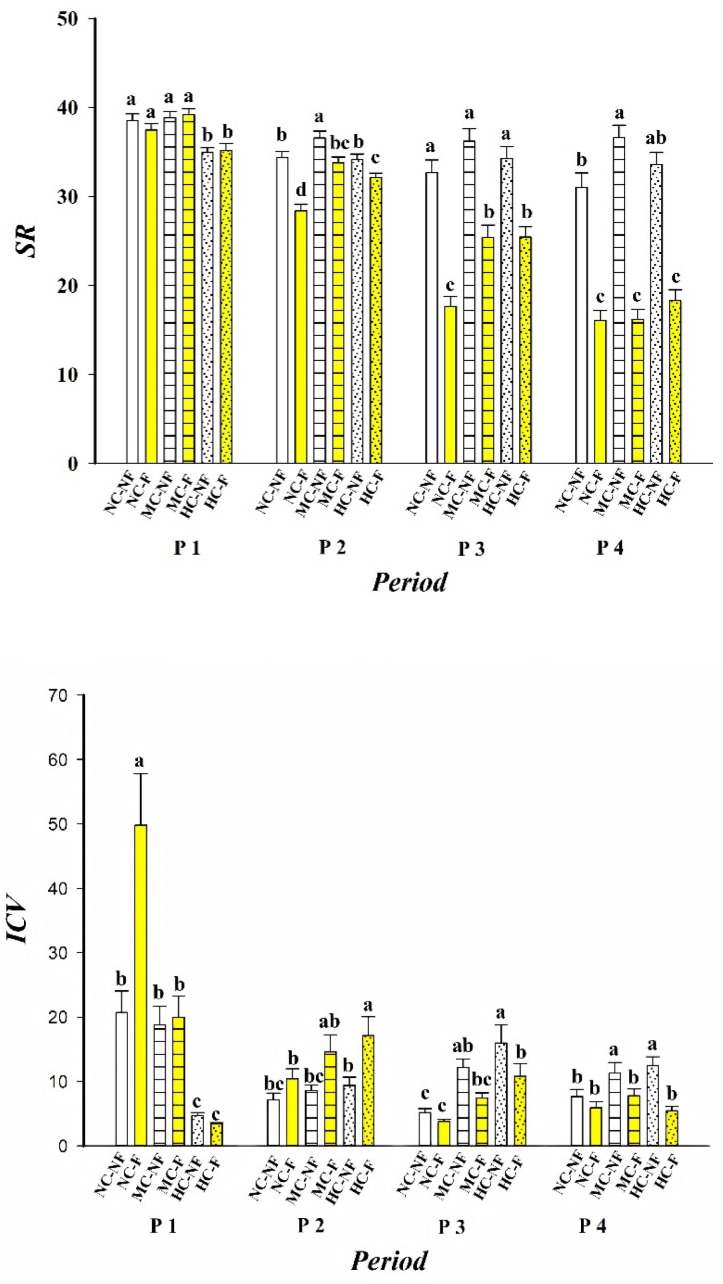
Variance analysis of SR and ICV under different clipping and fertilization treatments. Species richness (SR) and community temporal stability (inverse of the coefficient of variation, ICV) under different clipping and fertilization treatments across four experimental periods (P1–P4). NC, MC, and HC indicate no clipping, moderate clipping, and heavy clipping level. NF and F indicate no fertilization and fertilization. Within each period, treatments labelled with the same lowercase letter are not significantly different from each other (*p* > 0.05), whereas treatments with different letters differ significantly (*p* < 0.05).

Analysis of species richness (SR) and community temporal stability (inverse of the coefficient of variation, ICV) across different periods revealed that both SR and ICV varied significantly under contrasting clipping and fertilization regimes (*p* < 0.05). Across all experimental periods, SR differed significantly among treatments. By contrast, ICV did not vary significantly with clipping treatment alone, nor was the clipping × fertilization interaction significant. However, the interaction between treatment and period was significant for both SR and ICV (Table 1; Figure 1).

Period I: In most clipping treatments (with the exception of HC-NF), ICV was positively correlated with SR, whereas fertilization exhibited no discernible effect.

Period II: A negative correlation between ICV and SR was observed across all treatments, suggesting that the combined effects of fertilization and clipping during this phase may have driven convergence towards a common stability mechanism.

Period III: Negative correlations persisted in unfertilized communities (NC-NF and MC-NF). In contrast, fertilized or clipped treatments (e.g., HC-F and MC-F) predominantly showed positive correlations, indicating that fertilization can reverse the direction of the relationship between stability and species richness.

Period IV: With the sole exception of the fertilized, non-clipped treatment (NC-F), all communities exhibited positive ICV-SR correlations. This pattern suggests that long-term clipping may amplify the positive effect of species richness on community stability, whereas fertilization counteracts this benefit (Table 2).

Collectively, these results imply that clipping, particularly sustained over the long term, may promote community stability (or reduce variability) by maintaining or increasing species richness and mitigating diversity loss. In contrast, fertilization consistently reduced species richness and was frequently associated with a shift towards negative ICV-SR correlations, suggesting that fertilization may destabilize communities or increase their variability.

### 2.2. Asynchrony Effect

Analysis of the species asynchrony index across treatments revealed that asynchrony effects were present in all communities throughout all experimental periods and temporal intervals (Table 3). Overall, fertilization exerted relatively weak and inconsistent effects on asynchrony. In most cases, asynchrony indices were comparable between fertilized (F) and corresponding unfertilized (NF) treatments. However, during the later stages of the experiment (Periods III and IV), fertilization exhibited a tendency to reduce asynchrony, particularly when combined with clipping. This suggests that long-term fertilization, especially when coupled with disturbance, may modestly attenuate the asynchronous fluctuation patterns among species.

In contrast to fertilization, clipping played a more positive role in maintaining high levels of asynchrony. Under unfertilized (NF) conditions, asynchrony indices remained consistently high (generally >0.98) across all four periods, indicating that, in the absence of fertilization, clipping disturbance helps sustain and potentially strengthen interspecific asynchronous responses.

During Periods I and II, asynchrony indices were uniformly high across all treatments (>0.976), with minimal variation among treatments. This indicates that the asynchrony effect served as a dominant and shared stability mechanism across communities during the early-to-mid experimental stages. By Periods III and IV, however, treatment-related divergence emerged. Asynchrony declined markedly in non-clipped (NC) treatments, irrespective of fertilization status.

Over the long term, the absence of disturbance (non-clipping) may lead to synchronized species responses, thereby weakening this critical stability buffering mechanism. Sustained clipping treatment effectively prevents such synchronization. Species asynchrony represents a fundamental and robust buffering mechanism that enables alpine meadow communities to withstand environmental fluctuations and is universally present across diverse treatments. Clipping serves as an effective treatment tool for maintaining high asynchrony, particularly under unfertilised conditions. Fertilization exerts a modest negative effect on asynchrony, an effect that becomes more pronounced over the long term and when combined with clipping.

### 2.3. Portfolio Effect

Regression analyses conducted across different periods and treatment combinations revealed that the portfolio effect, which is defined here as the variance–scaling relationship indicative of statistical averaging, was present only under specific conditions (Table 4). Across all periods, the portfolio effect occurred intermittently and with relatively low consistency in unfertilized treatments (NC-NF, MC-NF, and HC-NF). By contrast, fertilization consistently suppressed the effect: in nearly every fertilized treatment, the portfolio effect was abolished in at least one, and often multiple, time intervals.

Collectively, these results imply that clipping, particularly sustained over the long term, may promote community stability (or reduce variability) by maintaining or increasing species richness and mitigating diversity loss. In contrast, fertilization consistently reduced species richness and was frequently associated with a shift towards negative ICV-SR correlations, suggesting that fertilization may destabilize communities or increase their variability.

### 2.4. Selection Effect

Dominance rankings identified the three most abundant species in each of the six treatment communities, with R1 denoting the dominant species and R2 and R3 the subdominants (Table 5). Throughout the entire experimental period (Periods I–IV), the coefficients of variation of both the single dominant species (R1) and the combined top three dominants (R1R2/R1R2R3) differed highly significantly from those of the remaining subordinate species (OS) in the vast majority of cases (*p* < 0.001) (Table 6).

During Periods III and IV, however, fertilization began to markedly attenuate and, in some instances, reverse the contrast in fluctuation regimes between dominant and non-dominant species. In striking opposition, unfertilized clipping treatments, particularly MC-NF and HC-NF, sustained highly significant dominance effects throughout the experiment (all tests *p* < 0.001) (Table 7).

These results demonstrate that fertilization progressively dismantles the classical community stability structure in which dominant species exhibit lower population fluctuations than other species. By contrast, under unfertilized conditions, clipping serves to preserve this structure. The interplay between fertilization and clipping thus governs the degree of dynamic segregation in functional groups (dominant vs. subordinate species) within the community.

### 2.5. Interspecific Interactions

Analysis of the log response ratio (LnRR) across different periods revealed that interspecific interactions were present in all treatment communities throughout the experiment, but their nature shifted markedly depending on period and treatments (Table 8).

### 2.6. Structural Equation Modelling

**Figure 2:**
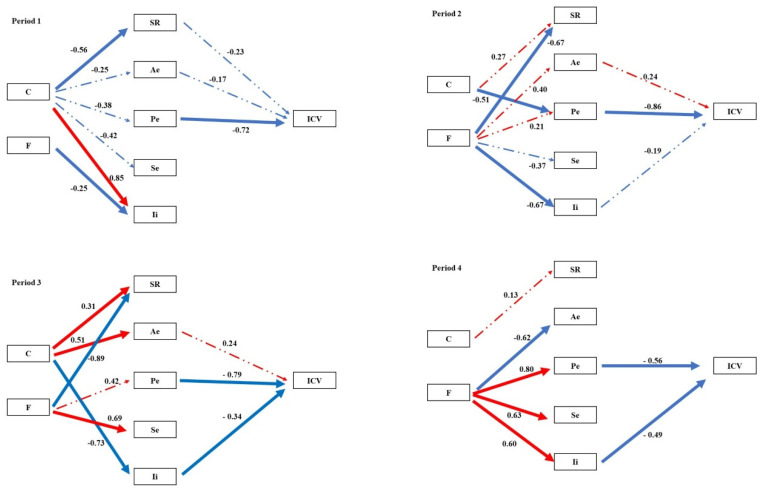
Path analysis of community temporal stability and mechanisms in different periods. Note: ICV is the temporal stability of a community. C represents clipping. F, fertilization; SR, species richness; Ae, asynchronous effect; Pe, portfolio effect; Se, selection effect; Ii, interspecific interaction. Red indicates a positive correlation, and blue indicates a negative correlation. Solid lines denote statistically significant relationships between connected variables, whereas dashed lines indicate non-significant relationships. The values above each path represent the standardized path coefficients, with positive and negative signs indicating the direction of the effect.

**Figure 3:**
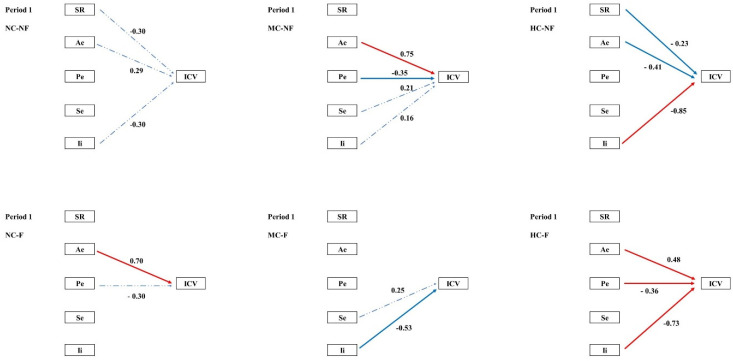
Path analysis of community temporal stability and mechanisms in period 1; Note: ICV is the temporal stability of a community; SR, species richness; Ae, asynchronous effect; Pe, portfolio effect; Se, selection effect; Ii, interspecific interaction. Red indicates a positive correlation, blue indicates a negative correlation, a solid line indicates significance, and a dotted line indicates non-significance.

**Figure 4:**
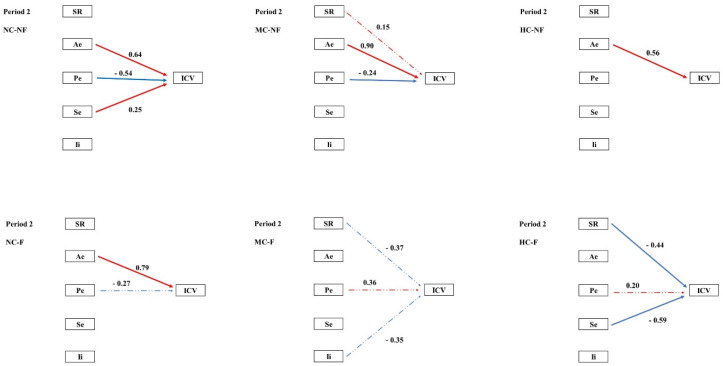
Path analysis of community temporal stability and mechanisms inperiod 2 Note: ICV is the temporal stability of a community; SR, species richness; Ae, asynchronous effect; Pe, portfolio effect; Se, selection effect; Ii, interspecific interaction. Red indicates a positive correlation, blue indicates a negative correlation, a solid line indicates significance, and a dotted line indicates non-significance.

**Figure 5:**
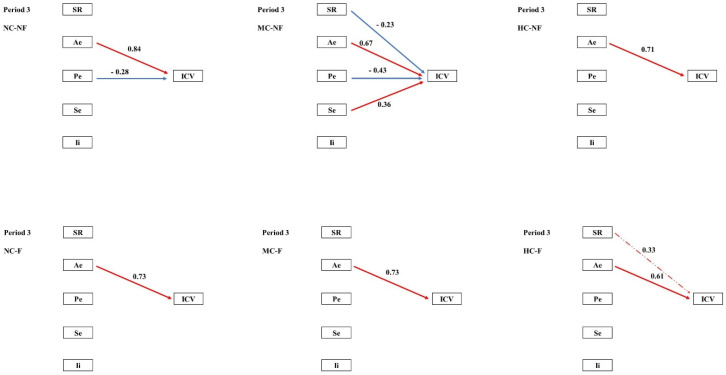
Path analysis of community temporal stability and mechanisms in period 3. Note: ICV is the temporal stability of a community; SR, species richness; Ae, asynchronous effect; Pe, portfolio effect; Se, selection effect; Ii, interspecific interaction. Red indicates a positive correlation, blue indicates a negative correlation, a solid line indicates significance, and a dotted line indicates non-significance.

**Figure 6:**
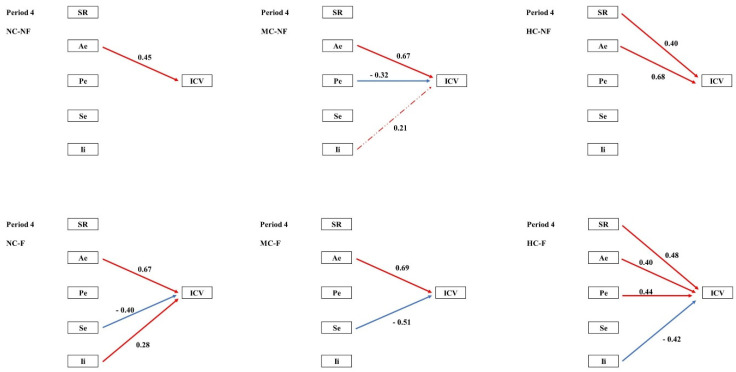
Path analysis of community temporal stability and mechanisms in period 4. Note: ICV is the temporal stability of a community; SR, species richness; Ae, asynchronous effect; Pe, portfolio effect; Se, selection effect; Ii, interspecific interaction. Red indicates a positive correlation, blue indicates a negative correlation, a solid line indicates significance, and a dotted line indicates non-significance.

Structural equation modelling (SEM) was employed to examine the pathways through which clipping and fertilization influenced species diversity and multiple stability-maintaining mechanisms, and how these in turn regulated community temporal stability (ICV) across the four experimental periods. In the pooled community analysis, the portfolio effect exerted a significant negative effect on ICV during Periods I–II. During Periods III–IV, both the portfolio effect and interspecific interactions contributed significant negative effects on ICV (Figure 2). SEM further revealed that the coexistence and fluctuation of stability mechanisms varied across both periods and treatments. (Figure 3, Figure 4, Figure 5 and Figure 6, Table A3).

## 3. Discussion

### 3.1. Differential Regulation of Species Diversity and Stability by Clipping and Fertilization

Human activities profoundly influence the species composition and dynamics of grassland communities through alterations in land use and resource inputs [17]. This study demonstrates that clipping (C) and fertilization (F) exert independent and opposing effects on species richness (SR) in alpine meadows: fertilization (F) significantly reduced SR, whereas clipping (C), particularly moderate clipping (MC), substantially increased SR (Figure 1). This pattern is consistent with the intermediate disturbance hypothesis [7,21], which posits that moderate disturbance sustains high diversity by alleviating competitive exclusion among dominant species, whereas high-intensity resource inputs often precipitate biotic homogenization and biodiversity loss [22,23].

Although a substantial body of theoretical and empirical work supports a positive correlation between species diversity and ecosystem stability [7,24,25], this relationship is highly context-dependent. In recovering grasslands or systems subjected to high nutrient inputs, for instance, the positive association may weaken, disappear, or even reverse [26,27]. In the present study, we similarly found that the relationship between SR and community temporal stability (ICV) varied dynamically across treatment combinations and experimental periods, rather than remaining constant. This aligns with findings from certain alpine meadow studies, which report that while high nitrogen addition reduces SR, it does not necessarily destabilize communities [28], suggesting that the operation of compensatory mechanisms is independent of diversity per se. These observations support the view that the diversity–stability relationship may decouple at community versus population levels [29], and underscore the limitations of relying solely on SR as a predictor of ecosystem stability. Elucidating how treatments modulate stability through intermediary ecological mechanisms is therefore of critical importance.

### 3.2. Co-Maintenance of Community Stability by Multiple Mechanisms and Their Responses to Treatments

Our results demonstrate that these mechanisms can operate independently or concurrently within communities, and that their relative importance is strongly modulated by clipping (C) and fertilization (F), exhibiting pronounced temporal dynamics.

The asynchrony effect (Ae) functioned as a foundational stability mechanism, persisting across all treatments and experimental periods (Table 3). Theoretical frameworks posit that asynchronous species responses to environmental fluctuations constitute a core mechanism buffering community productivity [20,24,30,31]. However, our structural equation models (SEMs) revealed that Ae promoted stability primarily indirectly by driving the portfolio effect (Pe) whereas its direct pathway to stability was non-significant (Figure 2).

The structural equation modeling results revealed that the direct path from the asynchrony effect (Ae) to community temporal stability (ICV) was not significant. At first glance, this finding appears to contradict classical theory, which generally posits that species asynchrony is one of the most direct mechanisms stabilizing community biomass. First, from an ecological mechanism perspective, the contribution of asynchrony to community stability inherently depends on its ability to translate into a statistical attenuation of community biomass variance, namely, the portfolio effect. In other words, the portfolio effect serves as the ‘statistical pipeline’ through which asynchrony exerts its stabilizing influence. When this pipeline variable is included in the model, the direct residual effect of asynchrony on stability (the portion not mediated by the pipeline) becomes negligible. Second, we cannot entirely rule out the influence of methodological limitations. Structural equation modeling is sensitive to sample size. Although the overall model fit was satisfactory, the relatively limited sample size may have reduced the statistical power to detect a small direct effect path. In summary, while our data did not support a direct effect of asynchrony on stability, this does not negate its importance in maintaining stability. We suggest that, within the specific disturbance context of this study (fertilization and clipping), the role of asynchrony was largely integrated into and manifested through the portfolio effect. Future research with larger sample sizes or meta-analytic approaches could further validate the generality of this relationship.

The portfolio effect (Pe) exhibited marked sensitivity to both treatment and time. Although previous studies have suggested that Pe plays a limited role in uneven communities [32] or is not a dominant stabilizing force in alpine meadows [33], we found that Pe exerted a significant direct stabilizing effect (negative effect on ICV) during the early experimental stages (Periods I–II). Notably, fertilization (F) amplified the stabilizing function of Pe under specific treatments (e.g., HC–F in Period I). This finding contrasts with studies reporting that fertilization reduces species asynchrony [34] or disrupts spatial asynchrony [22], and may reflect the unique compensatory dynamics of species in our system under resource input.

The roles of the selection effect (Se) and interspecific interactions (Ii) became increasingly prominent over the course of the experiment. According to the mass ratio hypothesis, ecosystem functioning is predominantly determined by the traits of dominant species [35]; consequently, the stability of these dominants is critical for overall community stability. However, sensitivity analyses examining the consequences of systematically altering subordinate species abundances revealed that non-dominant species also contribute to community stability, albeit through pathways distinct from the selection effect. While the removal or fluctuation of non-dominant species had negligible direct impact on aggregate biomass, consistent with the mass ratio hypothesis, it significantly modulated the strength and sign of interspecific interactions (Ii) among the remaining species. Our results show that long-term fertilization (F) progressively blurred the distinction in fluctuation regimes between dominant and subordinate species (Table 5), thereby potentially eroding the classical selection effect. Concurrently, fertilization consistently transformed intense interspecific competition into weak interactions (Table 8). Theory predicts that such weak interaction networks effectively dampen population fluctuations and enhance community stability [36]. Our SEM path analyses confirmed that Ii emerged as a key terminal mechanism influencing ICV during the later experimental stages (Figure 6). The shift in the nature of interspecific interactions (Ii) thus constitutes a central nexus linking treatments to community stability outcomes.

Although the asynchrony and portfolio effects observed in this study are universal stabilizing mechanisms, their relative importance may shift across different biomes. Compared with other ecosystems (e.g., tropical savannas or temperate abandoned farmlands), the alpine meadow on the Tibetan Plateau is characterized by unique low-temperature constraints, a short growing season, and slow soil development. Consequently, the specific thresholds for stability mechanism shifts (e.g., soil nitrogen content boundaries) likely vary depending on regional biodiversity and environmental contexts.

### 3.3. Dynamic Interplay of Stability Mechanisms Shapes Community Stability Across Successional Stages

The most penetrating insights of this study emerged from the network of mechanistic pathways revealed by structural equation modelling (SEM; Figure 2). Our results demonstrate that clipping (C) and fertilization (F) indirectly shape the long-term stability (ICV) of communities by “reprogramming” both the causal relationships among distinct stability mechanisms and the directionality of their effects.

During the early experimental phase, treatments modulated species richness (SR) and interspecific interactions (Ii), thereby driving a relatively parsimonious stability pathway centred on the Ae → Pe → ICV cascade. This pattern aligns with the classical theoretical framework in which biodiversity enhances stability through an insurance effect [25,37]. Over time, however, this pathway network underwent marked divergence. We observed that fertilization (F) tended to simplify the path network. By Period III, the strong positive forcing of SR on Pe had become exceptionally robust in fertilized treatments (Figure 2). This indicates that, although fertilization reduces absolute species numbers, the remaining species richness is crucial for sustaining the efficacy of the key stabilizing mechanism, the portfolio effect. This finding offers a novel perspective on the role of biodiversity in fertilized systems. This simplification of the pathway network under fertilization suggests that resource enrichment not only alters individual mechanisms but fundamentally restructures the causal architecture linking biodiversity to stability, a finding with important implications for predicting ecosystem responses to eutrophication.

Clipping (C), particularly at high intensity (HC), instead complexified the pathway network. In treatments such as HC–NF, multiple mechanisms (SR, Pe, and Se) frequently exerted contrasting effects on ICV (Figure 2). This suggests that intense disturbance forces communities to rely on multiple, sometimes mutually trade-off, stability strategies; under such conditions, community stability may become critically dependent on delicate balances among specific combinations of mechanisms [38,39].

By the late experimental stage (Period IV), prolonged exposure to contrasting treatments had given rise to sharply divergent stability solutions. Long-term high-intensity clipping (e.g., HC-NF and HC-F) established significant positive pathways, whether direct or indirect, from SR to ICV, underscoring the irreplaceable long-term insurance value of species diversity under persistent, severe disturbance [24,30]. Long-term fertilization, by contrast, appeared to reinforce the centrality of pathways involving interspecific interactions (Ii), suggesting that in resource-enriched environments, the nature and network structure of interspecific interactions assume progressively greater importance for the maintenance of stability.

### 3.4. Beyond Species Richness: Clipping and Fertilization Reconfigure Stability-Mechanism Networks to Determine Community Temporal Stability

This study demonstrates that the temporal stability (ICV) of alpine meadow communities is sustained by a multi-mechanism system in which the asynchrony effect (Ae) provides a universal foundation, the portfolio effect (Pe) and interspecific interactions (Ii) function as dynamically dominant core, and the selection effect (Se) offers conditional support. Clipping (C) and fertilization (F) act as critical environmental filters: rather than determining stability directly, they reshape the “configuration modes” and “synergistic pathways” of these intrinsic stability mechanisms through species filtering and the modification of resource regimes.

Our findings help reconcile long-standing debates on the diversity–stability relationship by demonstrating that this relationship is fundamentally dependent on the network of intermediary mechanisms modulated by treatments. Future research and grassland management should move beyond a narrow focus on species richness (SR) as a single indicator. Instead, adaptive treatments such as the application of moderate clipping and the judicious control of fertilization should be employed to cultivate community functional structures that enable the synergistic operation of multiple stability mechanisms, including species asynchrony and benign interspecific interactions [2,30]. Such an approach is of both theoretical and practical importance for sustaining and enhancing the resilience of alpine grassland ecosystem services in the face of global environmental change.

### 3.5. Generalizability and Limitations of the Findings

The pronounced interannual climatic variability on the Qinghai–Tibet Plateau may have confounded the observed treatment effects on community stability. Although we lacked long-term meteorological data for specific plots, the inclusion of a watering treatment in our experimental design provided a means to test for potential effects of moisture variability. We found that watering had no significant main effect on plant cover or ramet density in the early stages of the experiment, nor did it interact significantly with the clipping or fertilization treatments. Within the range of variability covered by this study, water availability was not a primary driver of the observed dynamics (Table A2). Although the asynchrony and portfolio effects observed in this study are considered universal stabilizing mechanisms in theoretical ecology, their relative importance and the specific thresholds at which they operate may shift across different biomes. Compared with other ecosystems (e.g., tropical savannas or temperate abandoned farmlands), the alpine meadow on the Tibetan Plateau is characterized by unique low-temperature constraints, a short growing season, and slow soil development. Consequently, the specific thresholds for stability mechanism shifts (e.g., soil nitrogen content boundaries) likely vary depending on regional biodiversity and environmental contexts. For instance, the strong mediating role of the portfolio effect observed here might be less pronounced in species-poor systems or more pronounced in systems with larger species pools. Therefore, while our core finding—that multiple stability mechanisms operate synergistically and are re-configured by disturbance and resource inputs—is likely generalizable, the quantitative relationships (e.g., the exact z-values or path coefficients) are probably context-dependent. Future research comparing similar experimental designs across different climatic zones and community types is needed to establish the boundary conditions of our conclusions.

It should be noted that, due to the exploratory nature of this study involving regression analyses across multiple treatment-time combinations, we did not correct for multiple comparisons. Therefore, this part of the analysis is exploratory in nature, and the reported *p*-values are primarily intended to reveal potential trends rather than serve as rigorous hypothesis tests, which may increase the probability of Type I errors.

When interpreting the findings of this study, several methodological limitations should be considered. First, the sample size for each treatment-period combination was relatively small for SEM, which may affect the stability of parameter estimates and the statistical power to detect small effect sizes, such as the direct path from Ae to ICV. Second, the series of regression analyses involving multiple treatment-period combinations falls within the scope of exploratory data analysis; we did not apply strict corrections for multiple comparisons, meaning the reported *p*-values are primarily intended to reveal potential trends. Third, the pronounced interannual climatic variability on the Qinghai–Tibet Plateau may have confounded the observed treatment effects. Although our watering experiment suggested that water availability was not a primary driver (Table A2), unmeasured factors like temperature variability may have contributed to the significant year effects. Finally, our study was conducted in a single alpine meadow site; multi-site replicated experiments are needed to confirm the generality of the observed mechanistic networks.

## 4. Materials and Methods

### 4.1. Study Area

The experiment was conducted from 2007 to 2018 in an alpine meadow dominated by *Kobresia humilis* at the Haibei Alpine Grassland Ecosystem National Field Research Station, Qinghai Province. The site is located on the northeastern Qinghai–Tibet Plateau (37°29′–37°45′ N, 101°12′–101°23′ E), at an elevation of 3200–3600 m [40]. The study area was a natural alpine meadow community at the establishment of the experiment in 2007, with no history of grazing. Currently, the region is designated as a prospective protected area with seasonal grazing, representing a transition zone between natural and semi-natural states. The mean annual temperature is −1.7 °C and the mean annual precipitation is 562 mm. The area is traditionally grazed by livestock during the winter–spring period (1 November to 31 May) [40].

### 4.2. Experimental Design and Sampling

Three clipping intensities were applied at the main-plot level, corresponding to the removal of 0%, 50%, and 70% of aboveground biomass, simulating no, moderate, and heavy grazing, respectively. For the MC and HC treatments, the stubble heights of the aboveground plant parts after clipping were 3 cm and 1 cm, respectively, while the NC treatment was not clipped. Fertilization was applied at the subplot level as either fertilized or unfertilized. The fertilizers used were urea and diammonium phosphate. The periods were divided based on the gradient changes in nitrogen addition rates. The four periods were 2007–2009, 2010–2012, 2013–2015, and 2016–2018, with the annual addition rates of nitrogen and phosphorus for each period being 1.64 g·m^−2^·y^−1^ and 0.50 g·m^−2^·y^−1^, 3.92 g·m^−2^·y^−1^ and 1.21 g·m^−2^·y^−1^, 4.38 g·m^−2^·y^−1^ and 1.34 g·m^−2^·y^−1^, and 20.00 g·m^−2^·y^−1^ and 8.00 g·m^−2^·y^−1^, respectively. The nitrogen addition rates for the four treatments were equivalent to 2.26, 5.40, 6.03, and 27.55 times the ambient wet nitrogen deposition flux at the Qinghai–Tibet Plateau positioning station (0.772 g·m^−2^·y^−1^), and 2.12, 5.08, 5.67, and 25.91 times the wet nitrogen deposition rate at the research station, respectively [41].

The fertilization rate increased progressively with each stage but remained constant within each three-year period. To prevent the lateral movement of nutrients, each subplot was isolated by inserting four iron sheets (2 m × 0.25 m) into the soil along its perimeter. Fertilized subplots received 4.6 g m^−2^ urea (20.4% N) and 1.10 g m^−2^ diammonium phosphate (5.9% N and 28.0% P). Data were collected annually in mid-August. In this study, the fertilization treatment involved the simultaneous addition of nitrogen and phosphorus, which precludes us from completely disentangling their independent contributions to community stability. However, multiple lines of evidence suggest that nitrogen likely played a dominant role in driving the observed declines in stability. Pre-experimental soil baseline analysis confirmed that plots assigned to different fertilization treatments did not differ significantly in baseline available phosphorus or total phosphorus. This rules out the alternative explanation that pre-existing differences in soil phosphorus could account for the observed changes in stability (Table A1).

### 4.3. Data Calculations

#### 4.3.1. Temporal Stability

Community temporal stability was quantified as the inverse of the coefficient of variation (*ICV*) of total community cover:

(1) $$ICV=\frac{µ}{\sigma }$$

where µ represents the temporal mean of total community cover and σ denotes the temporal standard deviation.

#### 4.3.2. Species Richness

Species richness (*SR*) was calculated as the total number of species present in each sampling plot:

(2) SR=S

where S denotes the total number of species recorded per plot.

#### 4.3.3. Asynchrony Effect

Species asynchrony was quantified as 1 − φb, where φb represents the synchrony of species fluctuations:

(3) $$1-\varphi b=1-\frac{\sigma^{2}}{{\left({\sum_{i}^{S}\sigma }_{i}\right)}^{2}}$$

where σ^2^ denotes the temporal variance of total community cover, and σ_i_ represents the temporal standard deviation of cover for species i. A value of 1 − φ_b_ = 1 indicates complete asynchrony, whereas 1 − φ_b_ = 0 signifies complete synchrony among species.

#### 4.3.4. Portfolio Effect

The portfolio effect was assessed by examining the scaling relationship between the temporal variance and mean cover of individual species. For each species within each treatment plot, we calculated the temporal variance (σ^2^) and the temporal mean cover (m). A power function of the form σ^2^ = c·m^z^ was fitted to the log-transformed data:

(4) σ^2^ = c·m^z^

Taking the logarithm of both sides yields

(5) Log(σ^2^) = z × log(m) + log(c)

σ^2^ denotes the temporal variance of species cover, and m represents the temporal mean of species cover.

The intensity of the portfolio effect is quantified by the exponent Z in the variance–mean power law relationship: z > 1 indicates that species variance increases more slowly than the mean, signifying the presence of a statistical averaging effect, with larger, with larger Z values reflecting a stronger portfolio effect

#### 4.3.5. Selection Effect

The selection effect was evaluated following the approach of Downing et al. Dominant species were identified based on dominance indices. For each of the six treatment communities, we first conducted paired-sample *t*-tests to compare the temporal coefficients of variation (CV) of cover between the dominant species and all subordinate species combined, thereby assessing whether dominant species exhibited intrinsically higher stability. Subsequently, we regressed the CV of dominant species cover against the CV of total community cover over the temporal series. A positive correlation between these two coefficients of variation was interpreted as evidence for the operation of a selection effect within the community.

#### 4.3.6. Interspecific Interactions

The strength and nature of interspecific interactions were quantified using the log response ratio (LnRR), following Downing et al. [42]:

In long-term field positioning experiments, directly isolating and quantifying root competition processes without disturbing the community is not feasible. Therefore, the log response ratio (LnRR) of aboveground biomass is used as an estimate of the net effect of neighbor removal. This method is widely accepted in community ecology for inferring the net effects of interspecific interactions. Although LnRR cannot be equated with a physiological competition coefficient, it integrates the cumulative outcomes of both aboveground and belowground interactions, effectively reflecting the net effect of neighbor presence at the community level, following McCann et al. [36].

(6) $$LnRR={In(R}_{0}/R_{w})$$

where R_w_ and R_0_ represent aboveground biomass in clipped and non-clipped treatment plots, respectively. The magnitude of |LnRR| reflects interaction intensity, with larger absolute values indicating stronger weak interactions [42].

We used the log response ratio of In (R_0_/R_w_) as the corresponding indicator and considered it as an inference of the net effect of removing neighbouring species, rather than a direct measure of the competition coefficient. In this study, the interaction intensity is inferred based on net changes in biomass, rather than representing a direct physiological competition coefficient.

### 4.4. Statistical Analyses and Structural Equation Modelling

The full dataset spanning 2007–2018 was partitioned into four discrete periods at three-year intervals following the approach of Liu [34]. To test the effects of clipping (C), fertilization (F), and their interaction on species richness (SR) and community stability (ICV) across the four experimental periods, we used a repeated-measures ANOVA. This approach was chosen to account for the non-independence of observations collected from the same permanent plots over multiple years. Prior to analysis, we assessed the assumptions of normality (Shapiro–Wilk test) and homogeneity of variances (Levene’s test). Data that did not meet these assumptions were log-transformed to improve conformity. Given that our time series consisted of four discrete periods, we evaluated the sphericity assumption using Mauchly’s test. When sphericity could be assumed (*p* > 0.05), we reported the results from the univariate repeated-measures ANOVA; when it was violated (*p* < 0.05), we applied the Greenhouse–Geisser correction to adjust the degrees of freedom and mitigate the risk of Type I error. All statistical analyses were conducted in SPSS (version 22.0, IBM Corp., Armonk, NY, USA), and figures were generated using SigmaPlot (version 12.5, Systat Software, Inc., San Jose, CA, USA).

Structural equation modelling (SEM) was implemented in AMOS 26.0 to disentangle the direct and indirect pathways through which clipping and fertilization influence community temporal stability (ICV). Species richness, asynchrony effect, portfolio effect, selection effect, and interspecific interactions were incorporated as key intermediate variables. Candidate models were screened using the Specification Search algorithm. We also used block as a random effect in our SEMs to control for the design structure. The optimal model was selected based on the following goodness-of-fit criteria, all of which indicate acceptable alignment between the model and observed data: χ^2^ test *p* > 0.05, comparative fit index (CFI) > 0.90, incremental fit index (IFI) > 0.90, and root mean square error of approximation (RMSEA) < 0.08. Standardized path coefficients and their significance levels were subsequently used to quantify the relative contributions of each ecological mechanism to community stability.

When interpreting the findings of this study, the following methodological limitations should be considered. First, the sample size for each treatment-period combination in this study was relatively small, which is at the lower end of the recommended sample size for structural equation modeling. Although all models met conventional goodness-of-fit criteria and we employed bootstrap resampling to enhance the robustness of parameter estimates, the relatively small sample size may still affect the stability of parameter estimates and the statistical power to detect small effect sizes. Therefore, caution should be exercised when interpreting the absolute values of specific path coefficients.

It should be noted that the series of regression analyses involving multiple treatment-period combinations in this study falls within the scope of exploratory data analysis. Therefore, we did not apply strict corrections for multiple comparisons. The primary purpose of these analyses is to identify potential trends and generate hypotheses, rather than to serve as confirmatory tests.

## 5. Conclusions

Research on natural communities in the Qinghai–Tibet Plateau has demonstrated that clipping can mitigate the loss of species diversity, whereas fertilization significantly reduces it. Our findings challenge the oversimplified ‘more species = good’ paradigm by demonstrating that stability emerges from the synergistic interplay of multiple mechanisms, whose relative importance shifts dynamically with environmental context and time. The asynchrony effect serves as a ubiquitous foundation for stability; the portfolio effect emerges as a critical positive driver in the early experimental stages. Over time, shifts in the nature of interspecific interactions (i.e., diminished competition) and alterations in selection effects gradually become pivotal in influencing long-term stability. Fertilization tends to reinforce the pathway of the portfolio effect and foster the formation of weak interactions, while clipping increases the complexity of the mechanisms maintaining stability. The portfolio effect and interspecific interactions constitute the primary factors sustaining temporal community stability across different years. In the long term, various stability mechanisms in differently treated communities independently or jointly sustain temporal stability. These findings provide a theoretical basis for the adaptive management of grassland ecosystems under global change: management strategies should move beyond the singular goal of maximizing species richness and instead focus on cultivating community functional structures that enable the synergistic operation of multiple stability mechanisms through moderate disturbance (e.g., moderate clipping) and judicious control of nutrient inputs.

## Figures and Tables

**Table 1:** ANOVA of SR and ICV in different periods.

Source of Variance	df	SR		ICV	
		*F*	*p*	F	*p*
C	2	21.486	0.000 *	2.799	0.062
B	2	3.956	0.020	0.597	0.551
C × B	4	0.553	0.697	1.103	0.355
F	1	386.614	0.000 *	2.719	0.100
C × F	2	2.674	0.070	0.612	0.543
P	3	156.646	0.000 *	15.311	0.000 *
C × P	6	5.692	0.000 *	16.229	0.000 *
F × P	3	84.603	0.000 *	7.446	0.000 *
C × F × P	6	2.541	0.020 *	2.281	0.035 *

C, clipping; B, block; F, fertilization; P, period; ×, interaction; df, degrees of freedom; *, *p* < 0.05.

**Table 2:** Regression of ICV and SR in different periods.

Period	Treatments	Regression Equations	R^2^	F-Test	*p*
I	NC-NF	y = 0.4499x + 2.1041	0.010	0.048	0.963
	NC-F	y = 2.0527x − 27.103	0.034	−0.264	0.795
	MC-NF	y = −0.5451x + 39.954	0.019	1.042	0.313
	MC-F	y = 1.9916x − 53.84	0.055	−0.791	0.441
	HC-NF	y = −0.2879x + 14.748	0.087	1.804	0.090
	HC-F	y = 0.0832x + 0.6466	0.092	0.281	0.782
II	NC-NF	y = −0.0063x + 7.388	0.000	0.600	0.557
	NC-F	y = −0.4312x + 22.667	0.045	1.590	0.131
	MC-NF	y = −0.0927x + 11.969	0.007	1.146	0.269
	MC-F	y = −1.3373x + 59.929	0.121	1.896	0.077
	HC-NF	y = −0.2633x + 17.926	0.014	0.945	0.359
	HC-F	y = 2.059x − 49.057	0.113	0.706	0.490
III	NC-NF	y = −0.0546x + 6.967	0.017	1.995	0.063
	NC-F	y = 0.1231x + 1.6138	0.139	1.157	0.264
	MC-NF	y = −0.0571x + 14.202	0.004	1.639	0.121
	MC-F	y = 0.3025x − 0.2197	0.301	−0.073	0.943
	HC-NF	y = 0.3502x + 4.248	0.026	0.223	0.826
	HC-F	y = 0.3502x + 4.248	0.026	−0.356	0.727
IV	NC-NF	y = 0.256x − 0.346	0.154	−0.070	0.945
	NC-F	y = −0.1199x + 7.8247	0.020	2.257	0.038
	MC-NF	y = 0.2948x + 0.5358	0.063	0.051	0.960
	MC-F	y = 0.012x + 7.5817	0.000	1.879	0.079
	HC-NF	y = 0.5854x − 7.9407	0.232	−0.859	0.403
	HC-F	y = 0.11x + 4.0584	0.019	1.083	0.295

NC, non-clipping; MC, moderate clipping; HC, heavy clipping; F, fertilization; NF, non-fertilization.

**Table 3:** Asynchronous effect index of different treatments in different intervals.

Period	Communities	Asynchronous Effect Index
I	NC-NF	0.994 ± 0.005
	NC-F	0.996 ± 0.010
	MC-NF	0.993 ± 0.007
	MC-F	0.994 ± 0.010
	HC-NF	0.976 ± 0.031
	HC-F	0.987 ± 0.015
II	NC-NF	0.983 ± 0.024
	NC-F	0.987 ± 0.014
	MC-NF	0.981 ± 0.014
	MC-F	0.990 ± 0.010
	HC-NF	0.981 ± 0.023
	HC-F	0.990 ± 0.010
III	NC-NF	0.956 ± 0.029
	NC-F	0.950 ± 0.030
	MC-NF	0.987 ± 0.009
	MC-F	0.966 ± 0.035
	HC-NF	0.985 ± 0.006
	HC-F	0.976 ± 0.015
IV	NC-NF	0.980 ± 0.013
	NC-F	0.956 ± 0.037
	MC-NF	0.987 ± 0.019
	MC-F	0.944 ± 0.037
	HC-NF	0.987 ± 0.009
	HC-F	0.965 ± 0.030

Note: NC, non-clipping; MC, moderate clipping; HC, heavy clipping; F, fertilized; NF, non-fertilized.

**Table 4:** Regression parameters of the power-law relationship between species variance and mean under different treatments and periods.

Period	Treatments	Regression Equations	Z	95% CI	R2	F-Test	*p*
I	NC-NF	y = −4.892x − 1.456	−4.892	[−18.23, 8.45]	0.091	0.702	0.430
	NC-F	y = 6.961x − 1.121	6.961	[−8.45, 22.37]	0.147	1.208	0.308
	MC-NF	y = −5.509x − 1.664	−5.509	[−11.42, 0.40]	0.411	4.877	0.063
	MC-F	y = 4.097x − 0.965	4.097	[−10.12, 18.32]	0.059	0.441	0.528
	HC-NF	y = 3.314x − 1.097	3.314	[0.71, 5.91]	0.564	9.047	0.020 *
	HC-F	y = 2.499x − 1.079	2.499	[0.63, 4.37]	0.589	10.042	0.016 *
II	NC-NF	y = 2.559x − 1.006	2.559	[0.81, 4.31]	0.625	11.647	0.011 *
	NC-F	y = 2.167x − 0.997	2.167	[−0.21, 4.55]	0.396	4.588	0.069
	MC-NF	y = 4.980x − 0.712	4.980	[2.21, 7.75]	0.707	16.927	0.004 *
	MC-F	y = 4.222x − 1.105	4.222	[1.23, 7.21]	0.606	10.752	0.014 *
	HC-NF	y = 1.360x − 1.384	1.360	[−0.79, 3.51]	0.240	2.208	0.181
	HC-F	y = 0.254x − 1.489	0.254	[−2.87, 3.37]	0.005	0.036	0.856
III	NC-NF	y = 0.979x − 1.483	0.979	[−0.87, 2.83]	0.179	1.524	0.257
	NC-F	y = 1.510x − 0.921	1.510	[0.26, 2.76]	0.532	7.949	0.026 *
	MC-NF	y = −0.332x − 1.473	−0.332	[−8.24, 7.58]	0.001	0.009	0.927
	MC-F	y = −0.334x − 1.558	−0.334	[−3.62, 2.96]	0.007	0.051	0.828
	HC-NF	y = −0.532x − 1.596	−0.532	[−2.22, 1.16]	0.070	0.528	0.491
	HC-F	y = −0.004x − 1.381	−0.004	[−2.76, 2.75]	0.000	0.000	0.998
IV	NC-NF	y = 1.815x − 1.243	1.815	[−0.47, 4.11]	0.335	3.522	0.103
	NC-F	y = −0.429x − 1.121	−0.429	[−0.96, 0.10]	0.335	3.522	0.103
	MC-NF	y = 1.433x − 1.338	1.433	[−2.31, 5.17]	0.106	0.834	0.391
	MC-F	y = 3.065x − 0.767	3.065	[−1.53, 7.67]	0.265	2.522	0.156
	HC-NF	y = −0.042x − 1.531	−0.042	[−1.79, 1.71]	0.000	0.003	0.958
	HC-F	y = 0.895x − 1.600	0.895	[−1.45, 3.25]	0.109	0.855	0.386

NC, non-clipping; MC, moderate clipping; HC, heavy clipping; F, fertilization; NF, non-fertilization. *, *p* < 0.05. The intensity of the portfolio effect is quantified by the slope z. A value of z > 1 indicates the presence of the portfolio effect (the variance of species increases more slowly than the mean), with larger z values reflecting a stronger effect.

**Table 5:** Dominant species in different treatment communities in each period.

Period		NC-NF	NC-F	MC-NF	MC-F	HC-NF	HC-F
I	R1	*S. aliena*	*S. aliena*	*S. aliena*	*S. aliena*	*S. pulchra*	*S. pulchra*
	R2	*S. nigrescens*	*S. nigrescens*	*S. nigrescens*	*P. annua*	*O. baxoiensis*	*S. aliena*
	R3	*S. pulchra*	*K. humilis*	*S. pulchra*	*K. humilis*	*S. aliena*	*S. nigrescens*
II	R1	*S. nigrescens*	*S. nigrescens*	*S. pulchra*	*S. pulchra*	*S. pulchra*	*S. pulchra*
	R2	*S. aliena*	*S. pulchra*	*S. nigrescens*	*K. humilis*	*K. humilis*	*K. humilis*
	R3	*S. pulchra*	*E. nutans*	*L. nanum*	*S. nigrescens*	*S. aliena*	*S. nigrescens*
III	R1	*S. nigrescens*	*S. nigrescens*	*S. pulchra*	*E. nutans*	*S. pulchra*	*E. nutans*
	R2	*S. pulchra*	*E. nutans*	*L. nanum*	*S. pulchra*	*L. nanum*	*S. pulchra*
	R3	*S. aliena*	*S. pulchra*	*S. nigrescens*	*S. nigrescens*	*T. mongolicum*	*S. nigrescens*
IV	R1	*S. nigrescens*	*E. nutans*	*S. pulchra*	*E. nutans*	*S. pulchra*	*E. nutans*
	R2	*S. pulchra*	*S. nigrescens*	*S. nigrescens*	*S. pulchra*	*L. nanum*	*S. pulchra*
	R3	*M. chinensis*	*A. flaccidus*	*L. nanum*	*S. nigrescens*	*O. ochrocephala*	*S. nigrescens*

I, II, III, and IV refer to different periods; NC, non-clipping; MC, moderate clipping; HC, heavy clipping; F, fertilized; NF, non-fertilized. R1, R2, R3, dominant species.

**Table 6:** Student’s *t* test of variation coefficient of dominant species and other species.

Period	Communities	R1 & OS		R1R2 & OS		R1R2R3 & OS	
		*t*-Value	*p*	*t*-Value	*p*	*t*-Value	*p*
I	NC-NF	13.342	0.000 *	12.762	0.000 *	10.853	0.000 *
	NC-F	18.961	0.000 *	11.855	0.000 *	10.247	0.000 *
	MC-NF	14.495	0.000 *	14.074	0.000 *	11.652	0.000 *
	MC-F	13.236	0.000 *	15.695	0.000 *	14.520	0.000 *
	HC-NF	18.761	0.000 *	14.927	0.000 *	14.826	0.000 *
	HC-F	16.261	0.000 *	12.408	0.000 *	12.530	0.000 *
II	NC-NF	15.527	0.000 *	11.903	0.000 *	7.603	0.000 *
	NC-F	14.544	0.000 *	9.892	0.000 *	7.029	0.000 *
	MC-NF	13.859	0.000 *	11.910	0.000 *	11.257	0.000 *
	MC-F	13.108	0.000 *	8.404	0.000 *	5.111	0.000 *
	HC-NF	15.325	0.000 *	12.897	0.000 *	10.933	0.000 *
	HC-F	13.041	0.000 *	10.113	0.000 *	7.836	0.000 *
III	NC-NF	17.512	0.000 *	13.529	0.000 *	8.656	0.000 *
	NC-F	9.301	0.000 *	4.032	0.000 *	1.237	0.222
	MC-NF	13.655	0.000 *	8.967	0.000 *	5.764	0.000 *
	MC-F	4.561	0.000 *	1.767	0.083	3.833	0.000 *
	HC-NF	18.585	0.000 *	10.579	0.000 *	6.453	0.000 *
	HC-F	10.426	0.000 *	1.864	0.068	2.590	0.012 *
IV	NC-NF	17.469	0.000 *	5.309	0.000 *	0.068	0.946
	NC-F	5.247	0.000 *	1.196	0.237	1.240	0.220
	MC-NF	15.642	0.000 *	10.398	0.000 *	8.221	0.000 *
	MC-F	0.451	0.654	4.196	0.000 *	6.224	0.000 *
	HC-NF	15.519	0.000 *	9.362	0.000 *	5.955	0.000 *
	HC-F	40.059	0.000 *	13.548	0.000 *	9.479	0.000 *

NC, non-clipping; MC, moderate clipping; HC, heavy clipping; F, fertilization; NF, non-fertilization; R1, R2, R3, dominant species; OS, other species; *, *p* < 0.05.

**Table 7:** Regression of dominant species R1 and variation coefficients of other species coverage in different treatment communities.

	Communities	Regression Equations	R^2^	F-Test	*p*
I	NC-NF	y = 0.186x + 0.614	0.015	0.249	0.624
	NC-F	y = 0.288x + 0.595	0.036	0.595	0.452
	MC-NF	y = 1.080x− 0.016	0.965	439.696	0.000 *
	MC-F	y = 1.154x − 0.073	0.965	417.426	0.000 *
	HC-NF	y = 1.371x − 0.083	0.082	1.434	0.249
	HC-F	y = 0.770x − 0.294	0.055	0.924	0.351
II	NC-NF	y = 0.230x + 0.499	0.011	0.173	0.683
	NC-F	y = 0.107x + 0.072	0.199	3.983	0.063
	MC-NF	y = 1.176x + 0.064	0.351	8.647	0.010 *
	MC-F	y = 0.954x + 0.207	0.151	2.838	0.111
	HC-NF	y = −0.024x + 0.382	0.000	0.003	0.961
	HC-F	y = 0.199x+ 0.314	0.010	0.159	0.695
III	NC-NF	y = 1.555x + 0.072	0.254	5.444	0.033 *
	NC-F	y = 0.062x + 0.434	0.001	0.013	0.910
	MC-NF	y = 0.102x + 0.238	0.003	0.049	0.828
	MC-F	y = 1.512x + 0.562	0.108	1.932	0.184
	HC-NF	y = 0.066x + 0.318	0.001	0.013	0.909
	HC-F	y = 0.571x + 0.498	0.063	1.074	0.316
IV	NC-NF	y = −0.044x + 0.325	0.001	0.017	0.897
	NC-F	y = 0.270x + 0.585	0.079	1.375	0.258
	MC-NF	y = 2.175x + 0.068	0.201	4.016	0.062
	MC-F	y = −0.356x + 0.505	0.003	0.052	0.823
	HC-NF	y = 0.253x+ 0.208	0.024	0.394	0.539
	HC-F	y= −0.843x + 0.568	0.056	0.942	0.346

NC, MC, and HC indicate no cutting, moderate cutting, and heavy clipping level. NF and F indicate no fertilization and fertilization, *, *p* < 0.05.

**Table 8:** Interaction index of different treatment communities.

Period	Communities	Interaction Index
I	NC-NF	0.006 ± 0.111
	NC-F	−0.052 ± 0.082
	MC-NF	0.094 ± 0.133
	MC-F	0.010 ± 0.107
	HC-NF	0.180 ± 0.241
	HC-F	0.178 ± 0.285
II	NC-NF	0.025 ± 0.240
	NC-F	−0.081 ± 0.180
	MC-NF	−0.031 ± 0.164
	MC-F	−0.201 ± 0.121
	HC-NF	0.090 ± 0.229
	HC-F	−0.172 ± 0.127
III	NC-NF	0.037 ± 0.288
	NC-F	0.105 ± 0.320
	MC-NF	−0.311 ± 0.097
	MC-F	−0.198 ± 0.166
	HC-NF	−0.175 ± 0.131
	HC-F	−0.233 ± 0.167
IV	NC-NF	0.016 ± 0.184
	NC-F	0.059 ± 0.345
	MC-NF	−0.201 ± 0.112
	MC-F	−0.031 ± 0.207
	HC-NF	−0.110 ± 0.147
	HC-F	0.104 ± 0.226

Note: I, II, III, and IV refer to different periods; NC, non-clipping; MC, moderate clipping; HC, heavy clipping; F, fertilized; NF, non-fertilized.

## Data Availability

Data will be made available upon request to the corresponding author, as the raw data are currently part of ongoing research projects. We intend to deposit them in a public repository upon completion of the relevant studies.

## Abbreviations

The following abbreviations are used in this manuscript:

ICV	Temporal stability of community
C	Clipping
F	Fertilization
P	Period
SR	Species richness
Ae	Asynchronous effect
Pe	Portfolio effect
Se	Selection effect
Ii	Interspecific interaction

## Appendix A

**Table A1:** Univariate Analysis of Variance (Two-way ANOVA) for Differences in Soil Nutrients Measured Before the Experiment Commenced in 2007 Among Experimental Treatments.

Sources of Variance	df	Organic Matter		Total Nitrogen		Nitrate Nitrogen		Ammonium Nitrogen		Available Nitrogen		Available Phosphorus		Total Phosphorus		Available Nitrogen to Phosphorus Ratio	
	m, n	F	*p*	F	*p*	F	*p*	F	*p*	F	*p*	F	*p*	F	*p*	F	*p*
Clipping (C)	2, 2	1393.76	0.001 *	47.62	0.021 *	0.56	0.64	13.98	0.067	6.39	0.135	14.76	0.063	4.23	0.191	2.73	0.268
Block (B)	1, 2	73.1	0.013 *	0.01	0.923	2.24	0.273	2.32	0.267	3.24	0.214	8.79	0.097	0.17	0.723	7.65	0.11
Fertilization (F)	1, 15	0.84	0.373	1.06	0.32	1.29	0.273	2.23	0.156	2.26	0.154	3.67	0.075	0.53	0.479	0.73	0.407
C × B	2, 15	0.01	0.986	0.62	0.551	1.16	0.341	0.62	0.551	0.76	0.484	0.844	0.449	1.42	0.272	3.4	0.061
C × F	2, 15	1.49	0.255	3	0.08	1.58	0.239	0.69	0.217	2.09	0.158	2.04	0.165	0.22	0.802	1.18	0.336

C, clipping; B, block; F, fertilization; ×, interaction; df, degrees of freedom; *, *p* < 0.05.

**Table A2:** ANOVA for the effects of clipping, fertilizing, watering and the different years on the Elymus nutans.

Source of Variance	df	F(Cover)	p(Cover)
C	2	15.839	0.000 *
W	1	0.346	0.559
F	1	0.029	0.866
Y	1	4.144	0.047 *
C × Y	2	0.922	0.405
W × Y	1	0.352	0.556
F × Y	1	0.017	0.897
C × W	2	0.33	0.721
C × F	2	0.507	0.606
W × F	2	0.816	0.371
C × W × F	2	0.452	0.636
Y × C × W	2	1	0.375
Y × C × F	2	0.042	0.959
Y × W × F	1	1.321	0.256
Y × C × W × F	2	0.514	0.601

Note: C, clipping; F, fertilizing; W, watering; and Y, years; *, *p* < 0.05.

**Table A3:** Goodness-of-fit indices for SEMs across different periods and treatments.

Period	Treatments	Chi-Square	CFI	IFI	RMSEA
I	NC-NF	14.979	0.896	0.988	0.070
	NC-F	33.273	0.871	0.907	0.067
	MC-NF	9.546	1.000	1.071	0.001
	MC-F	21.118	0.897	0.975	0.072
	HC-NF	32.352	0.925	0.949	0.054
	HC-F	33.792	0.950	0.928	0.057
II	NC-NF	7.463	1.000	1.199	0.001
	NC-F	23.059	0.961	0.983	0.081
	MC-NF	17.442	0.946	0.858	0.062
	MC-F	27.629	0.949	0.944	0.076
	HC-NF	16.949	0.952	0.989	0.079
	HC-F	14.611	0.920	0.988	0.043
III	NC-NF	27.656	0.981	0.904	0.067
	NC-F	37.498	0.934	0.958	0.065
	MC-NF	12.368	0.917	0.933	0.035
	MC-F	31.324	0.903	0.916	0.016
	HC-NF	21.639	0.990	0.911	0.068
	HC-F	23.760	0.960	0.909	0.083
IV	NC-NF	21.063	0.992	0.957	0.065
	NC-F	20.100	0.949	0.960	0.075
	MC-NF	14.974	0.926	0.852	0.046
	MC-F	30.608	0.978	0.907	0.067
	HC-NF	25.315	0.967	0.995	0.080
	HC-F	17.964	0.985	0.933	0.073

Note: NC, non-clipping; MC, moderate clipping; HC, heavy clipping; F, fertilized; NF, non-fertilized; CFI (Comparative Fit Index), IFI (Incremental Fit Index), RMSEA (Root Mean Square Error of Approximation). Values of CFI and IFI closer to 1 indicate better model fit, while smaller RMSEA values (typically <0.08) suggest better fit.

**Table A4:** Species list of experimental plot in Qinghai Haibei.

Species	Family	Genus
*Leontopodium nanum*	*Compositae*	*Leontopodium*
*Lonicera minuta*	*Caprifoliaceae*	*Lonicera*
*Kobresia humilis*	*Cyperaceae*	*Kobresia*
*Carex scabrirostris*	*Cyperaceae*	*Carex*
*Geranium pratense*	*Geraniaceae*	*Geranium*
*Artemisia hedinii*	*Compositae*	*Artemisia*
*Taraxacum maurocarpum*	*Compositae*	*Taraxacum*
*Elymus nutans*	*Gramineae*	*Elymus*
*Galium echinocarpum*	*Rubiaceae*	*Galium*
*Gentiana aristata*	*Gentianaceae*	*Gentiana*
*Euphrasia regelii*	*Scrophulariaceae*	*Euphrasia*
*Potentilla anserina*	*Rosaceae*	*Potentilla*
*Potentilla bifurca*	*Rosaceae*	*Potentilla*
*Oxytropis kansuensis*	*Leguminosae*	*Oxytropis*
*Pedicularis kansuensis*	*Scrophulariaceae*	*Pedicularis*
*Tibetia himalaica*	*Leguminosae*	*Tibetia*
*Kobresia pygmaea*	*Cyperaceae*	*Kobresia*
*Glaux maritima*	*Primulaceae*	*Glaux*
*Bupleurum smithii*	*Umbelliferae*	*Bupleurum*
*Oxytropis ochrocephala*	*Leguminosae*	*Oxytropis*
*Medicago falcata*	*Leguminosae*	*Medicago*
*Astragalus membranaceus*	*Leguminosae*	*Astragalus*
*Ligularia virgaurea*	*Compositae*	*Ligularia*
*Ligularia sagitta*	*Compositae*	*Ligularia*
*Iris potaninii*	*Iridaceae*	*Iris*
*Gentiana aperta*	*Gentianaceae*	*Gentiana*
*Notopterygium forbesii*	*Umbelliferae*	*Notopterygium*
*Delphinium caeruleum*	*Ranunculaceae*	*Delphinium*
*Viola bulbosa*	*Violacea*	*Viola*
*Anaphalis hancockii*	*Compositae*	*Anaphalis*
*Gentiana straminea*	*Gentianaceae*	*Gentiana*
*Saussurea pulchra*	*Compositae*	*Saussurea*
*Ranunculus pulchellus*	*Ranunculaceae*	*Ranunculus*
*Elsholtzia densa*	*Labiatae*	*Elsholtzia*
*Ranunculus membranaceus*	*Ranunculaceae*	*Ranunculus*
*Ranunculus brotherusii*	*Ranunculaceae*	*Ranunculus*
*Gentiana pudica*	*Gentianaceae*	*Gentiana*
*Taraxacum mongolicum*	*Compositae*	*Taraxacum*
*Koeleria cristata*	*Gramineae*	*Koeleria*
*Medicago archiducis-nicolai*	*Leguminosae*	*Medicago*
*Pedicularis lyrata*	*Scrophulariaceae*	*Pedicularis*
*Aster flaccidus*	*Compositae*	*Aster*
*Lancea tibetica*	*Scrophulariaceae*	*Lancea*
*Anaphalis lactea*	*Compositae*	*Anaphalis*
*Iris goniocarpa*	*Amaryllidaceae*	*Iris*
*Saussurea nigrescens*	*Compositae*	*Saussurea*
*Parnassia trinervis*	*Saxifragaceae*	*Parnassia*
*Stellaria uda*	*Caryophyllaceae*	*Stellaria*
*Gentianopsis paludosa*	*Gentianaceae*	*Gentianopsis*
*Anemone obtusiloba*	*Ranunculaceae*	*Anemone*
*Scirpus distigmaticus*	*Cyperaceae*	*Scirpus*
*Draba nemorosa*	*Cruciferae*	*Draba*
*Microula sikkimensis*	*Boraginaceae*	*Microula*
*Polygonum sibiricum*	*Polygonaceae*	*Polygonum*
*Viola kunawarensis*	*Violaceae*	*Viola*
*Hypecoum leptocarpum*	*Papaveraceae*	*Hypecoum*
*Ajania tenuifolia*	*Compositae*	*Ajania*
*Silene gracilicaulis*	*Caryophyllaceae*	*Silene*
*Gentiana farreri*	*Gentianaceae*	*Gentiana*
*Rheum pumilum*	*Polygonaceae*	*Rheum*
*Potentilla nivea*	*Rosaceae*	*Potentilla*
*Potentilla parvifolia*	*Rosaceae*	*Potentilla*
*Taraxacum asiaticum*	*Compositae*	*Taraxacum*
*Festuca ovina*	*Gramineae*	*Festuca*
*Iris dichotoma*	*Iridaceae*	*Iris*
*Stipa aliena*	*Gramineae*	*Stipa*
*Morina chinensis*	*Dipsacaceae*	*Morina*
*Thalictrum rutifolium*	*Ranunculaceae*	*Thalictrum*
*Poa annua*	*Gramineae*	*Poa*
*Veronica ciliata*	*Scrophulariaceae*	*Veronica*
*Leontopodium longifolium*	*Compositae*	*Leontopodium*
*Thalictrum alpinum*	*Ranunculaceae*	*Thalictrum*
*Comastoma polycladum*	*Gentianaceae*	*Comastoma*
*Polygonum viviparum*	*Polygonaceae*	*Polygonum*
